# Ultralow Dose MSCT Imaging in Dental Implantology

**DOI:** 10.2174/1874210601812010087

**Published:** 2018-01-31

**Authors:** Gerlig Widmann, Asma'a A. Al-Ekrish

**Affiliations:** 1 Department of Radiology, Medical University of Innsbruck, Innsbruck, Austria; 2 Department of Oral Medicine and Diagnostic Sciences, College of Dentistry, King Saud University, Riyadh, Saudi Arabia

**Keywords:** Radiation dose, Dental Implantology, Cone beam computed tomography, Multislice computed tomography, Ultralow dose, Filtered backward projection

## Abstract

**Introduction:**

The Council Directive 2013/59 Euratom has a clear commitment for keeping medical radiation exposure as low as reasonably achievable and demands a regular review and use of diagnostic reference levels.

**Methods:**

In dental implantology, the range of effective doses for cone beam computed tomography (CBCT) shows a broad overlap with multislice computed tomography (MSCT). More recently, ultralow dose imaging with new generations of MSCT scanners may impart radiation doses equal to or lower than CBCT. Dose reductions in MSCT have been further facilitated by the introduction of iterative image reconstruction technology (IRT), which provides substantial noise reduction over the current standard of filtered backward projection (FBP).

**Aim:**

The aim of this article is to review the available literature on ultralow dose CT imaging and IRTs in dental implantology imaging and to summarize their influence on spatial and contrast resolution, image noise, tissue density measurements, and validity of linear measurements of the jaws.

**Conclusion:**

Application of ultralow dose MSCT with IRT technology in dental implantology offers the potential for very large dose reductions compared with standard dose imaging. Yet, evaluation of various diagnostic tasks related to dental implantology is still needed to confirm the results obtained with various IRTs and ultra-low doses so far.

## 1 INTRODUCTION

Cone beam computed tomography (CBCT) and multi slice computed tomography (MSCT) are essential imaging modalities in dental implantology. The importance of cross-sectional imaging in visualizing the amount and shape of the edentulous ridge, and determining the position of neurovascular structures, nasal cavity, and maxillary sinuses relative to proposed implant sites is reflected by current guidelines for dental and maxillofacial radiology such as the position statement of the American Academy of Oral and Maxillofacial Radiology [1], European Association of Osseointegration (EAO) devised consensus guidelines [2] and the evidence-based guidelines from the European sedentex CT project [3]. Multiplanar cross-sectional and volume rendering 3D images may be obtained from MSCT datasets and used to assess implant sites and plan and guide treatment assisted by dedicated software and computer-assisted fabrication of surgical guides.

However, the increasing use of cross-sectional imaging has become an increasing public and medical concern [4]. Radiologic societies have started awareness campaigns such as the Image Gently and Image Wisely Campaigns and the American College of Radiology dose index registry initiatives [5]. The European Union Council Directive 2013/59/Euratom of 5 December 2013 has set the basic safety standards for protection against the dangers arising from exposure to ionising radiation and requires a clear commitment to radiation protection. All doses due to medical exposures should be kept as low as reasonably achievable consistent with obtaining the required medical information; and the amount of patient exposure should form part of the report. This National Council on radiation protection has also attempted to emphasize the importance of optimization in medical imaging through modification of the ALARA As Low As Reasonably Achievable concept to ALADA, As Low As Diagnostically Achievable, which stresses that the aim of imaging should be to use the lowest possible dose which will produce diagnostic images [6]. The EU directive will have an impact on dental imaging and demands appropriate compliance by personnel involved in requesting, acquiring, and interpreting radiographic images. In addition, diagnostic reference levels for dental implant imaging should be developed and regularly reviewed.

The range of effective doses obtained from CBCT may show a broad overlap with MSCT. New generations of MDCT scanners have an enormous potential for dose reduction with ultralow dose imaging and may impart radiation doses equal to or lower than CBCT [7-9]. The purpose of this article is to review the current state of the art of ultralow dose MSCT imaging and Iterative Image Reconstruction Technology (IRT) in dental implantology imaging and to summarize their influence on spatial and contrast resolution, image noise, tissue density measurements, and validity of linear measurements of the jaws.

## 2 ULTRALOW DOSE IMAGING

There are many options for dose reduction including modification of basic imaging parameters, X-ray beam prefiltering, and application of IRT. Unfortunately, different MSCT vendors each have their own proprietary nomenclature for similar scanning factors, which may create confusion when comparing different scanners [5]. However, organizations must archive volume CT dose index (CTDI_vol_) and Dose Length Product (DLP) for every MSCT examination by series or anatomic area imaged. The CTDI_vol_ and DLP are estimates of average X-ray output from an acquired MSCT image series, which allows an easy comparison of the effect of different protocols, techniques and devices [10].

The most practical and most commonly used method to mitigate radiation exposure is to reduce tube voltage (kV) and effective tube current time product (mAs). High resolution reference protocols at 120 kV/100-200 mAs with a CTDI_vol_ of approximately 30 mGy can be replaced by low dose protocols using 100-110 kV/50-80 mAs with a CTDI_vol_ of approximately 10 mGy [7, 11]. With reduced mA, CT examination of the maxilla may be performed at an effective dose of 22 μSv, which is comparable to that of a dental panoramic radiograph (26 μSv), while a CT scan of the mandible may be performed at an effective dose of 123 μSv which is comparable to a full-mouth survey with intra-oral films (150 μSv) [12]. Considering a scan length of 10 cm (*i.e*. a dentoalveolar scan of mandible & maxilla), progressive reduction in the kV and mAs using ultralow dose protocols led to estimated effective doses of 87 µSv (CTDI_vol_ 4.14 mGy), 55 µSv (CTDI_vol_ 2.63 mGy), 21 µSv (CTDI_vol_ 0.99 mGy), and 11 µSv (CTDI_vol_ 0.53 mGy), which are marked reductions compared to the effective dose of 771 µSv imparted by 120 kV/100 mAs (CTDI_vol_ 36.71 mGy) [13].

X-ray tubes in MSCT usually allow voltages ranging from 140 kV down to 80 kV as the lowest possible tube potential. Recently, an X-ray tube providing 70 kV has been tested by comparing 70 kV/75 mAs (CTDI_vol_ 2.33 mGy) with 100 kV/40 mAs (CTDI_vol_ 3.95 mGy) and 120 kV/40 (CTDI_vol_ 6.31 mGy) for imaging of paranasal sinus [14]. Image noise was significantly higher at lower doses; however, the signal noise ratio of soft tissues was increased at 70 kV and showed no significant difference to 100 kV due to a higher organ attenuation of soft tissue structures with lower tube voltage settings [14]. Also, a 40% decrease in CTDI_vol_ and calculated effective dose was obtained with MSCT of the jaws when kV was reduced from 100 to 80, even though the mA was increased from 35 to 40 [9].

Another method for reducing MSCT dose is increasing the pitch. Pitch (beam pitch) is defined as the table movement per rotation/total thickness of all of the simultaneously acquired slices (total beam width (*n* x T), with *n* slices and slice thickness T) [15]. The CTDI_vol_ decreases linearly with increasing pitch, but the number of photons contributing to the images decreases also. A pitch of ≤ 1 is typically used in MSCT of bone to decrease helical interpolation artifacts [15]. Using a second-generation dual source MSCT (combined use of a 90° shifted X-ray tube) with large detector coverage for face and sinus imaging a pitch of 3 may provide sufficient diagnostic image quality compared with two single-source reference protocols with pitch 0.9 [16].

Prefiltration of the X-ray beam with a Tin filter has also been shown to reduce MSCT doses through constriction of the energy spectrum [17]. Tin filtration using 100 kV/ 250 mAs at CTDI_vol_ 2.02 mGy may be effective for trauma imaging and Tin filtration using 100kV/ 150 mAs at CTDI_vol_ 1.22 mGy may provide sufficient information at the lowest dose [17].

## 3 ITERATIVE RECONSTRUCTION TECHNOLOGY (IRT)

Recently, IRTs have been implemented to reduce image noise and improve quality of low dose images when compared with the traditionally used filtered back projection technique (FBP) [18]. Iterative reconstruction in image space (IRIS; Siemens Healthcare) includes an additional “correction loop” in which the obtained images are gradually approximated to their actual density distribution, thereby reducing image noise. The subjective image quality of a 60% dose reduced protocol at 120 kV/24 mAs with IRIS may demonstrate no significant difference to a reference protocol using 120 kV/60 mAs with FBP [19]. Sinogram-affirmed iterative reconstruction (SAFIRE; Siemens Healthcare) estimates the noise content in raw data by fluctuations in neighboring voxels and subtracts the noise stepwise in up to 5 repetitive correction loops. This technology may reduce image noise by 15% - 85% [20].

Adaptive statistical iterative reconstruction (ASIR; GE Healthcare) used the images obtained from the FBP algorithm but integrates a comparison of the pixel values with an ideal value to selectively identify and then subtract noise from an image at adaptive blend levels, which can be selected typically from 10% to 100% [21]. Model Based Iterative Reconstruction (MBIR, VEO; GE Healthcare) uses a more complex system of prediction models. It does not rely on the FBP as a starting point but integrates the noise and the spatial and geometric features of the x-ray beam and detector technology [22]. Unfortunately, MBIR requires a considerably long computational time and, therefore, may not be practical for emergency imaging. Also, MBIR is not available with a bone kernel. In a human cadaver, ultralow dose images at CTDI_vol_ 3.48 mGy, and 2.19 mGy (ASIR-100), and CTDI_vol_ 0.82 mGy (MBIR) may provide similar subjective bone image quality compared with the FBP reference at CTDI_vol_ 30.48 mGy [11]. In addition, 3D images may show similar subjective image quality at CTDI_vol_ 3.48 mGy (all reconstructions), 2.19 mGy (FBP and ASIR-100), 0.82 mGy (FBP, ASIR-100, and MBIR), 0.44 mGy (MBIR), and 0.22 mGy (MBIR) [23].

## 4 SPATIAL RESOLUTION

One study evaluated spatial and contrast resolution of ultralow dose images and ASIR and MBIR using the SedentexCT IQ Phantom (Leeds Test Objects Ltd., Boroughbridge, UK) [13]. As a general finding, reconstructions using the standard convolution kernel have a lower spatial resolution than those with bone kernel. Coronal sharpness is lower than axial sharpness, which reflects the non-isotropic image acquisition of MSCT. Also, with each reconstruction technique (FBP, ASIR, MBIR), lower doses produced images with lower spatial resolution. However, when a standard convolution kernel was used, ultralow dose protocols at CTDI_vol_ of 4.14 mGy and 2.36 mGy combined with MBIR showed comparable spatial resolution to the standard clinical reference protocol at CTDI_vol_ of 36.58 mGy using FBP; this may allow for a dose reductions of up to 93%. However, ASIR was not found to significantly improve spatial resolution over FBP, regardless of the kernel used.

In a cadaver, dislocated craniofacial fractures were clearly detected with protocols using a CTDI_vol_ as low as 1.0 mGy and non-dislocated fractures at a CTDI_vol_ of 2.6 mGy, regardless of the reconstruction technique used. As such, due to their image smoothing effects, ASIR and MBIR did not improve fracture detection over FBP [24].

## 5 CONTRAST RESOLUTION

Reductions in dose increase noise and decrease contrast resolution. Approximately 80% reduction in dose with FBP demonstrated a markedly reduced CNR but no alteration of the modulation transfer function of two MSCT devices [25].

When evaluating contrast noise ratio (CNR) using the SedentexCT IQ Phantom imaged with ultralow dose MSCT and IRTs, MBIR may show the highest CNR and retain the highest relative CNR, followed by ASIR 100, ASIR 50 and FBP (26). At CTDI_vol_ of 0.53 mGy MBIR limited CNR reduction to 55% compared with a 87% reduction using FBP [26]. CNR using ASIR-100 may be better than ASIR 50 and FBP but the results seem to be not statistically significant [26].

In another study, when a bone kernel was used (except with MBIR) at a CTDIvol of 0.53 mGy (98% of a standard dose), the use of ASIR and MBIR was found to reduce noise in images of the jaw bones compared to FBP; ASIR-50 reduced noise by 15%, ASIR-100 by 24% and MBIR by 38%. However, ASIR and MBIR did not appear to offer an advantage over FBP in this regards at higher doses [27]. Furthermore, the same study found that reconstruction technique had no significant effect on noise levels when a standard kernel was used with FBP and ASIR. On the other hand, studies of images of soft tissues and air obtained with standard kernels found that MBIR images demonstrated less noise than ASIR [11, 20, 28]. The contrasting results between images of bone and soft tissue may suggest that the effect of reconstruction technique and kernel on noise is not just affected by the dose level, but also by the type of tissue being imaged.

## 6 BONE DENSITY MEASUREMENTS

Bone density can be measured using the calibrated grey values as Hounsfield Units (HU), which are defined as linear transformations of measured X-ray attenuation coefficients of materials with reference to water [29]. The HU scale is based on two fixed values, which are 0 HU for water and -1000 HU for air. Unlike CBCT, HU are relatively consistent across different MSCT scanners and HU calibration is an integrated part of quality control. Reduced MSCT doses are known to produce altered tissue densities within the images [30]. Evaluation of ultralow dose imaging using the SedentexCT IQ Phantom showed that HU values may vary significantly at very low doses [26].

Standard kernels may show less HU variability than bone kernels and ultralow dose protocols using FBP may show errors of up to 273 HU [26]. Application of MBIR with standard kernel can reduce the error value to 138 HU for the CTDI_vol_ of 0.53 mGy [26]. In human cadaver jaw bones, decreasing doses show an increase in mean HU of the jaw bones. Maximum mean differences in HU of 178.35 (bone kernel) and 273.74 (standard kernel) may be observed when comparing several ultralow-dose protocols with a reference protocol. Use of MBIR tends to show a lower discrepancy in HUs with ultra-low doses than the other reconstruction techniques. Using ultralow-dose protocols at a CTDI_vol_ of 0.44 mGy and a standard kernel may demonstrate a difference of only 69.43 HU using MBIR compared with 162.48 HU using FBP. However, the same study did not detect a significant difference in density between the different reconstruction algorithms when a bone kernel was used [27].

## 7 CLINICAL SIGNIFICANCE OF HU AND DENSITY WITH ULTRA-LOW DOSE MDCT

The clinical impact of altered HU and noise profiles on implant imaging is still not completely clear. The image noise produced by a 90% reduction in dose combined with ASIR and MBIR did not significantly interfere with subjective image quality of 3D models [11]. But to fully understand how such ultra-low dose protocols may affect computer guided surgery, further studies on thresholding accuracy and CAD-CAM production of surgical guides need to be conducted.

The impact of the observed differences in noise and density between the various protocols on objective bone quality evaluation during implant site analysis is also still not clear. The HU obtained by standard dose MSCT has been correlated with primary implant stability, but there is still no reference standard to relate specific HU values to the determinants of implant site treatment planning and/or success [31-35]. Furthermore, since HU values vary with reductions in dose and reconstruction technique, any such standard, if developed, would only apply to images obtained with similar exposure protocols, especially the kV [30].

Regarding subjective classification of bone, the Lekholm and Zarb classification of bone assessed from standard dose MSCT images has been significantly correlated with subjective drilling resistance during implant osteotomies [36, 37]. Also, crestal cortical bone thickness assessed from standard dose MDCT has been strongly correlated with primary implant stability [32]. However, the changes in noise and HU seen with various combinations of low dose MSCT and reconstruction techniques, as well as the detrimental effect of MBIR on the visibility of thin bone may conceivably affect the subjective evaluation of bone [38]. Therefore, further studies are needed to relate the subjective appearance of bone from ultra-low dose images with the determinants of implant stability and success.

## 8 VALIDITY OF LINEAR MEASUREMENTS OF THE JAWS

Images obtained with MSCT at CTDI_vol_ 2.5 mGy may provide an acceptable accuracy of measurements for the purpose of maxillofacial surgery and image-based oral implant surgery [39]. With regards to linear measurements of dental implant sites recorded from sectional images, most combinations of ultra-low doses ranging from CTDI_vol_ 0.44-4.19 mGy combined with FBP, ASIR, and MBIR, demonstrated no systematic variation in linear measurements when compared with a standard dose FBP protocol (CTDI_vol_ 30.48-36.71 mGy). The only combinations which demonstrated a statistically significant difference were within ± 0.1 mm (95% CI ± 1.15mm) of the reference measurements, which is not clinically significant [9].

Evaluating the influence of ultralow doses on Target Registration Error (TRE) of navigated surgery of facial and cranial bones, a 98% reduction in MDCT dose down to a CTDI_vol_ of 0.76 mGy using three different CT scanners, including one intraoperative scanner, showed no significant adverse effect on TRE [23].

## CONCLUSION

Following public and medicolegal demands, efforts towards dose reduction have become an increasing responsibility in daily clinical routine. Implantologists and radiologists have to be aware that radiation dose of cross-sectional imaging may vary substantially depending on scan parameters, including kV, mAs, pitch, collimation and others. Modern MSCT has an enormous potential for dose reductions and currently allows doses less than CBCT. Lowering MSCT doses increases noise and HU. But the noise can be markedly reduced with the application of IRTs. Ultralow dose images at CTDI_vol_ markedly less than 1 mGy did not significantly affect the height and width measurements of implant sites or TREs of navigated surgery. Further studies are still needed to investigate the effect of ultra-low doses and IRTs on other diagnostic tasks related to dental implantology such as visualization of the inferior alveolar nerve, accuracy of 3D segmentation and evaluation of influence on CAD-CAM produced surgical guides to confirm the results obtained so far. Last- generation CT scanners using ultralow dose technology and IRTs should be introduced in further clinical studies, in conformance with the goals of the Euratom directive. Implementation of ultra-low doses in dental implantology may help in the formulation of DRLs to improve compliance with the ALADA concept.
